# Identification and genomic characterization of a novel porcine parvovirus in China

**DOI:** 10.3389/fvets.2022.1009103

**Published:** 2022-09-20

**Authors:** Yajing Guo, Guangzhi Yan, Shengnan Chen, Hui Han, Jiaming Li, Haoquan Zhang, Shicheng Luo, Mingjie Liu, Qingqing Wu, Qingxian Li, Changchun Tu, Liangzong Huang, Wenjie Gong

**Affiliations:** ^1^School of Life Science and Engineering, Foshan University, Foshan, China; ^2^Guangdong Findergene Biotechnology Co., Ltd, Foshan, China; ^3^Changchun Veterinary Research Institute, Chinese Academy of Agricultural Sciences, Changchun, China; ^4^State Key Laboratory for Zoonotic Diseases, Key Laboratory for Zoonoses Research, Ministry of Education, College of Veterinary Medicine, Jilin University, Changchun, China

**Keywords:** porcine parvovirus, genetic diversity, virome, China, prevalence

## Abstract

Porcine parvoviruses (PPVs) are a group of small non-enveloped viruses with seven species (porcine parvovirus 1–7, PPV1-7) have been identified. In this study, a novel porcine parvovirus, provisionally named porcine parvovirus 8 (PPV8), was initially identified *via* high-throughput sequencing (HTS) in porcine reproductive and respiratory syndrome virus-positive samples collected from swine herds in Guangdong province, 2021. The nearly full-length genome of PPV8 strain GDJM2021 is 4,380 nucleotides in length with two overlapping open ORFs encoding NS1 and VP1 respectively. Sequence analysis indicated that PPV8 shared 16.23–44.18% sequence identity at the genomic levels to PPV1-7 with the relatively highest homology to PPV1. PPV8-GDJM2021 shared 31.86–32.68% aa sequence identity of NS1 protein with those of PPV1 and porcine bufavirus (PBuV), and formed an independent branch neighboring to those formed by members of the genus *Protoparvovirus*. Of the 211 clinical samples collected from 1990 to 2021, 37 samples (17.5%) distributed over 12 regions in China were positive for PPV8 with time spanning 24 years (1998–2021). To our knowledge, this is the first report on the genomic characterization of the novel PPV8 and its epidemiological situations in China.

## Background

Members of family *Parvoviridae* are small, resilient, non-enveloped viruses with linear, single-stranded DNA genomes of 4–6 kb in size. Parvoviruses has two large open reading frames (ORFs) encoding non-structural protein (NS1) and capsid protein (VP) respectively (1). According to the viral host range, parvoviruses can be divided into the three subfamilies, *Densovirinae, Hamaparvovirinae* and *Parvovirinae*, and members of the latter two subfamilies infect vertebrate hosts (2). Nine genera of *Parvovirinae* have been defined with five out of them detected in pigs: *Protoparvovirus, Bocaparvovirus, Copiparvovirus, Chapparvovirus* and *Tetraparvovirus*.

Porcine parvovirus 1 (PPV1), a significant pathogen causing reproductive failure in pigs, which belongs to the genus *Protoparvovirus*, was first discovered in Germany in 1965 (3). In the past 20 years, a series of novel parvoviruses have been identified in pigs, including PPV2-7, which have also been detected in China. PPVs were classified into four genera of parvoviruses based on NS1 protein sequence homology: *Protoparvovirus* (PPV1), *Tetraparvovirus* (PPV2–PPV3), *Copiparvovirus* (PPV4–PPV6), *Chapparvovirus* (PPV7) (4–10). However, unlike PPV1, the pathogenicity of other PPVs is not clear. PPVs can be detected in multiple tissues and organs of pigs, such as sera, rectal swabs, nasal swabs, aborted fetuses and lung lavage (8, 9, 11), and PPV2 has a high prevalence in pig lung samples (11). Advances in metagenomic technology in recent years have greatly accelerated the identification of novel parvoviruses in both diseased and healthy animals, such as PPV7 was identified by metagenomic sequencing of pooled rectal swabs from adult pigs (9).

In the present study, we discovered a novel parvovirus named as PPV8 in the lungs of sick pigs by virome technology, which likely represent a novel species within the *Protoparvovirus* genus. Our study initially reveals the prevalence of PPV8 in pig herds from different regions in China.

## Methods

### Clinical samples

In this study, lung tissue samples of 55 sick pigs displayed high fever or respiratory syndromes were collected in 2021 from 55 different pig farms in Guangdong Province, China and these samples were confirmed as porcine reproductive and respiratory syndrome virus (PRRSV) positive by RT-qPCR as detected previously using methods established in our laboratory (details not shown). In addition, 156 clinical samples from sick pigs from 19 regions in China, including 115 ones positive for classical swine fever virus (CSFV) (12), which were taken between 1990 and 2021.

### Sample processing and high-throughput sequencing

To investigate DNA virus in the PRRSV-positive pigs, the 55 lung tissue samples were homogenized in Minimal Essential medium (MEM), followed by clarification through centrifugation at 12,000 × g, 4°C for 10 min. The obtained supernatant was filtered through a 0.45 μm filter and then subjected to total DNA extraction using DNeasy Blood & Tissue Kit (Qiagen, Germany). Sequencing library was generated using NEB Next^®^ Ultra™ DNA Library Prep Kit for Illumina (NEB, USA) according to the manufacturer's recommendations. Sequencing was performed on the Illumina high-throughput sequencing platform NovaSeq 6,000 (Novogene, China) and 150 bp paired-end reads were generated. The resulting raw reads were trimmed using Trimmomatic v0.39 (13), and pig genome sequence reads were removed from clean data by mapping against Sscrofa11.1 using Bowtie2 v2.4.1 (14). The remaining reads were de novo assembled into contigs using MEGAHIT v1.2.9 (15). The assembled contigs were aligned against a customized viral nucleotide reference database of GenBank (Taxonomy ID 10239) or the UniProt virus taxonomic database using blastn/blastx for searching viral sequences.

### Sequence alignment and phylogenetic analysis

Sequences alignments were performed using the MAFFT v7.470 program. Sequence comparison was conducted using BioAider v1.423 to analyze sequence identity of nucleotide (nt) and amino acid (aa) between PPV8 and other parvoviruses (16). A Maximum Likelihood (ML) tree was constructed using MEGA X software. Reliability of the ML tree was calculated using 1,000 bootstrap replicates. In addition to the PPV8, the complete sequences of several other parvoviruses were obtained from GenBank.

### PCR detection of PPV8 and sequencing

To investigate the prevalence of PPV8 infection in China, 211 clinical samples from sick pigs from 19 regions in China were tested for PPV8 by nPCR using PPV8-specific primers and PPV8-specific primers were designed based on contigs annotated to PPV8 in the virome. Total DNA extraction were performed as described above, and PCR amplification was performed with outer primer pair PPV8-outF: 5′- TGTTGGTTTGCACCTAGCG−3′/PPV8-outR: 5′- TGATGAGATGGTGGAACGC−3′ and inner primer pair PPV8-inF: 5′- TCCAAGTTGCCCTAGACAGC−3′/ PPV8-inR: 5′- GCCTCGTACATGTGGACCTC−3′. The thermal cycling conditions were 94°C for 3 min, followed by 35 cycles of 94°C for 30 s, 58°C for 30 s, 72°C for 30 s, and a final elongation step at 72°C for 10 min. Same conditions were used for nest-PCR amplification. The positive samples were sequenced and the fragment length was 554 bp. The PCR products were separated using 1% agarose gel electrophoresis and cloned into a pCE2 TA/Blunt-Zero vector (Vazyme, China). The positive clones sent to a commercial facility (GENEWIE, China) for sequencing.

### Virus isolation

Porcine kidney cell line (PK-15) and swine testicle cells (ST) frequently used to isolate PPV1 were used for *in vitro* isolation of PPV8 (17). PCR-positive samples were homogenized in minimal essential medium (MEM) and clarified at 10,000 × g for 10 min. After filtration *via* a 0.22 μm filter, the supernatant was inoculated onto cells and incubated for 2 h at 37°C and 5% CO_2_. Then the inoculum was discarded and MEM containing 5% fetal bovine serum (FBS) was added after washing two times. After incubation for 72 h, the cell cultures were harvested through three times of freezing and thawing and subsequently inoculated into cells for another four passages, and the cytopathic effects (CPEs) were monitored daily after inoculation, the cell cultures were also tested for PPV8 by nPCR.

## Results

### Virome analysis based high-throughput sequencing

Unbiased high-through sequencing was performed on Illumina MiSeq platform. A total of 43,323,656 raw reads from viral genome DNA. A total of 559 contigs from viral genome DNA were then generated by de novo assembly. The result shows that there are five kinds of vertebrate-associated viral families, which are *Adenoviridae, Anelloviridae, Circoviridae, Parvoviridae, Smacoviridae* (Table 1). These vertebrate-associated viral families were further annotated to nine genus and 11 species, such as Porcine circovirus 2 (PCV2), Torque teno sus virus (TTV), Porcine mastadenovirus B (PAdV), etc (Table 1). Twelve contigs were annotated to be parvovirus with one of unknow parvovirus (4,380 nt) showing low homology to the existing parvoviruses and blastp analysis of the 590-aa protein found 39.3% identity to bufavirus-3 (AB847989), indicating the possible presence of a novel parvovirus. Based on its homology to parvovirus and its host, the DNA virus was subsequently given the name porcine parvovirus type eight (PPV8). Furthermore, the whole genome of a PPV8 strain GDJM2021 was successfully amplified by PCR with the primer pairs, which are available upon request, and then sequenced by Sanger sequencing with ABI3730XL, the resulting genome sequence has been deposited in GenBank under accession number OP021638.

**Table 1:** The vertebrate-associated virus annotation results in DNA virome of pig lung.

**Family**	**Genus**	**Species**	**Contigs**	**Reads**
*Anelloviridae*	*Iotatorquevirus*	Torque teno sus virus 1b	77	145,060
	*Kappatorquevirus*	Torque teno sus virus k2a	6	24,638
*Circoviridae*	*Circovirus*	Porcine circovirus 2	50	2,106,163
*Smacoviridae*	*Porprismacovirus*	Porcine associated porprismacovirus	29	6,192
*Parvoviridae*	*Tetraparvovirus*	Porcine parvovirus 2	1	10
	*Copiparvovirus*	Porcine parvovirus 6	2	1,187
	*Chaphamaparvovirus*	Porcine parvovirus 7	3	37
	*Protoparvovirus*	Porcine Bufavirus	5	675
	*Protoparvovirus*	Unknown parvovirus	1	7,631
*Adenoviridae*	*Mastadenovirus*	Porcine mastadenovirus B	11	1,096

### Genome structure analysis of PPV8

**Figure 1:**
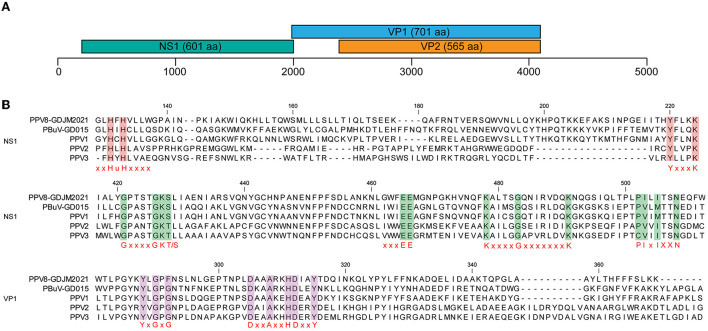
Genomic structure of PPV8-GDJM2021 **(A)** and multiple sequence alignment of the motif of PPV8-GDJM2021 with those of other parvoviruses **(B)**. **(A)** predicted ORFs encoding structural and non-structural proteins are indicated by boxes of different colors. The position and size of putative ORFs are indicated. **(B)** the conserved motifs in both the helicase domain in the NS1 proteins and the phospholipase A2 (PLA-2) domain in the VP1 proteins of PPV8-GDJM2021 and other porcine parvovirus, including, xxHuHxxxx (GLHFHVLLW) and YxxxK (YFLKK), Walker motifs (A, B, B', and C), calcium binding domain and enzymatic core motif.

The complete genome of PPV8-GDJM2021 was 4,380 nt in length and the G+C content was 45%, in which putative ORFs were predicted by ORF finder tool and then identified by blastp analysis. As a result, ORF1 is 1,806 nt encoding a putative NSP of 601 amino acids (aa), and ORF2 is 2,106 nt encoding a putative VP1 of 701 aa. ORF1 overlaps by 14 nt with ORF2, and a 565-aa VP2 is included in the structural protein VP1. In addition, the complete genome of PPV8-GDJM2021 contains a 242 nt 5'-untranslated region (UTR) and a 239 nt 3'-UTR at both ends (Figure 1A). Sequence comparison showed that NS1 protein contains several conserved motifs in the helicase domain, including ATP- or GTP-binding Walker A loop aa motif (GxxxxGKT/S; GPTSTGKS), and Walker B (xxxxEE; NLGWFEE), Walker B' (KxxxxGxxxxxxx K; KALTSGQNIRVDQK), and Walker C (PIxIXXN; PILITSN) aa motifs. In addition, NS1 protein also contains two conserved replication initiator (endonuclease) motifs, xxHuHxxxx (GLHFHVLLW) and YxxxK (YFLKK) (conserved aa are indicated at the bottom of the alignment (18) (Figure 1B). VP1 protein contains the putative catalytic residues (DxxAxxHDxxY; DAAARKHDIAYTD) of a phospholipase A2 (PLA2) domain (19). Furthermore, PPV8-GDJM2021 contains a calcium-binding loop (YLGPF) in the VP1 protein, rather than the “YXGXG” motif found in most parvoviruses (20) (Figure 1B).

Analysis of the NS1 protein sequences showed that PPV8-GDJM2021 is genetically distant from the known parvoviruses with highest sequence similarity (31.86-32.68%) to porcine bufavirus (PBuV, QDI06037.1) and PPV1 (NP_757369.1) (Table 2). According to the classification criteria for parvoviruses reported by International Committee on Taxonomy of Viruses (ICTV), the intra-genus aa sequence similarity of PPV NS1 protein is >30%, and the intra-species identity is >85% (1). Thus, PPV8-GDJM2021 represents a new species within the *Protoparvovirus* genus. Genomic sequence analysis indicated that PPV8-GDJM2021 and PPV1 (NC_001718.1) are relatively closely related to each other with 44.18% genome sequence identity, but are distant from PPV2-7 sharing only 16.23–24.17% sequence identity at the genomic level.

**Table 2:** Nucleotide and amino acid identities of PPV8-GDJM2021 with those of other parvoviruses.

**Genus**	**Virus**	**Genome size** **(nt)**	**PPV8-GDJM2021**		
			**Genome (nt)**	**NS1 (nt/aa)**	**VP1 (nt/aa)**
*Protoparvovirus*	Porcine parvovirus 1 (NC_001718.1)	5,075	44.18	43.90/31.86	45.33/33.12
	Porcine bufavirus strain GD015 (MK279317.1)	4,189	37.98	44.57/32.68	41.70/29.70
*Tetraparvovirus*	Parvovirus YX-2010/CHN (GU938300.1)	5,444	17.59	21.90/12.45	18.69/10.73
	Porcine hokovirus (EU200677.1)	5,114	18.07	22.15/12.48	19.54/11.49
*Copiparvovirus*	Porcine parvovirus 4 (GQ387499.1)	5,905	24.17	28.73/17.73	24.10/11.87
	Porcine parvovirus 5 (JX896318.1)	5,516	23.7	30.25/16.93	21.06/10.55
	Porcine parvovirus 6 (NC_023860.1)	6,148	17.82	24.91/15.41	16.23/8.29
*Chapparvovirus*	Porcine parvovirus 7 (KU563733.1)	4,103	16.23	17.73/9.08	15.98/6.78
*Dependoparvovirus*	Avian adeno-associated virus(AY186198.1)	4,694	20.1	24.68/17.66	27.29/16.95
*Bocaparvovirus*	Porcine bocavirus 3 (JF429834.1)	5,278	21.02	31.13/16.57	29.29/17.96
	Porcine bocavirus 1 (HM053693.2)	5,153	19.26	28.20/15.68	28.75/18.85
*Aveparvovirus*	Turkey parvovirus 260 (GU214706.1)	4,615	21.71	29.23/15.20	24.99/14.09
*Erythroparvovirus*	Pig-tailed macaque parvovirus (AF221123.1)	5,049	25.11	27.40/17.29	22.75/12.53
*Amdoparvovirus*	Skunk amdoparvovirus (KX981923.1)	4,242	33.08	34.07/20.03	35.26/24.28

### Phylogenetic analysis of PPV8

**Figure 2:**
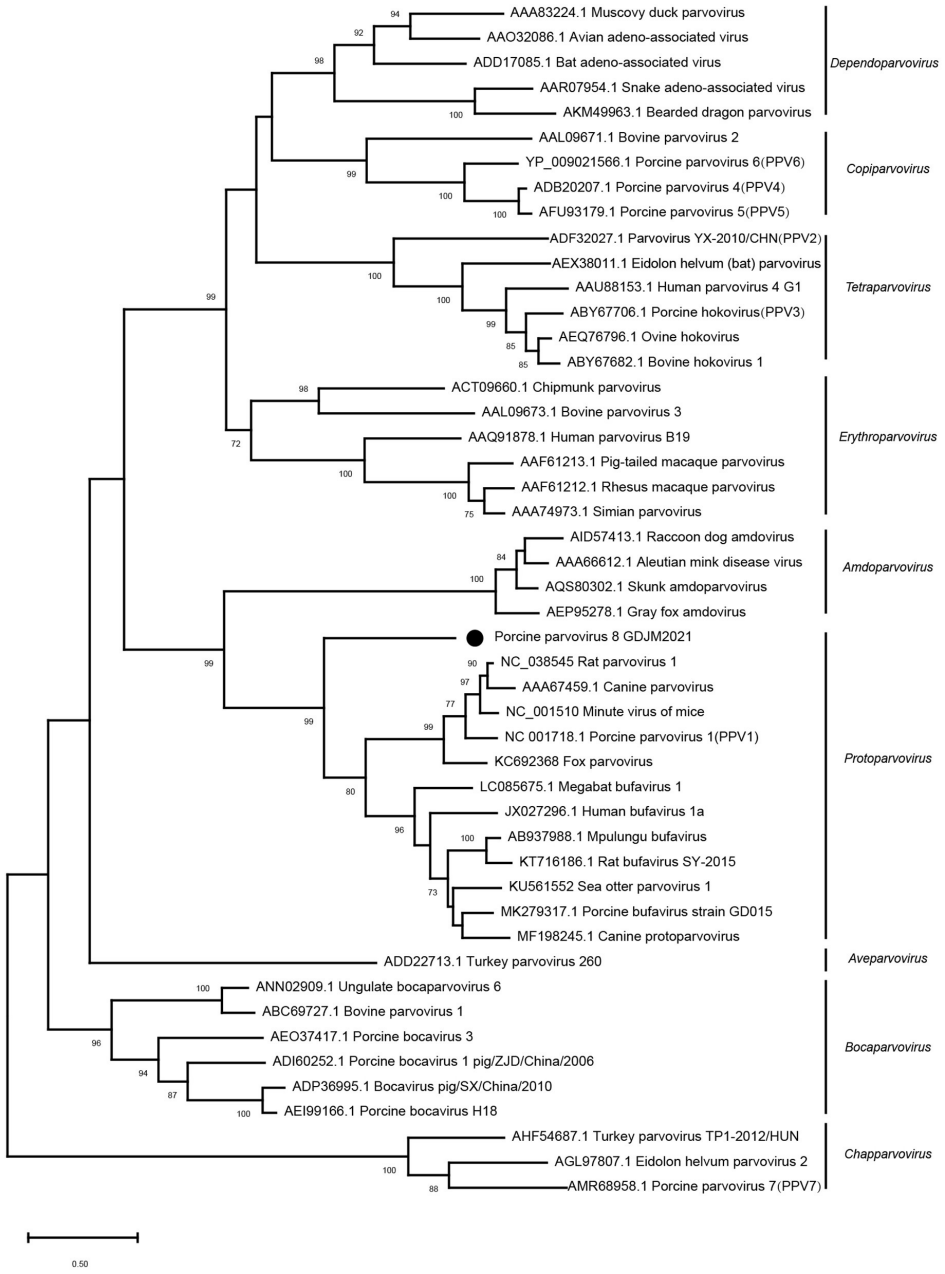
Phylogenetic trees of parvoviruses. Phylogenetic analysis was constructed based on full-length aa sequences of NS1 protein of PPV8-GDJM2021 and other reference strains of nine parvovirus genera retrieved from GenBank by MEGA X using the maximum-likelihood (ML) method and 1,000 bootstrap replicates.

Phylogenetic analysis based on the nucleotide sequences of NS1 was conducted with MEGA X using the maximum-likelihood method with 1,000 bootstrap replicates and a LG+G+I+F model. As shown in Figure 2, PPV8-GDJM2021 clustered together with the members belonging to the genus *Protoparvovirus*, but branched away from PPV2–7.

### Detection of novel parvovirus in China

**Figure 3:**
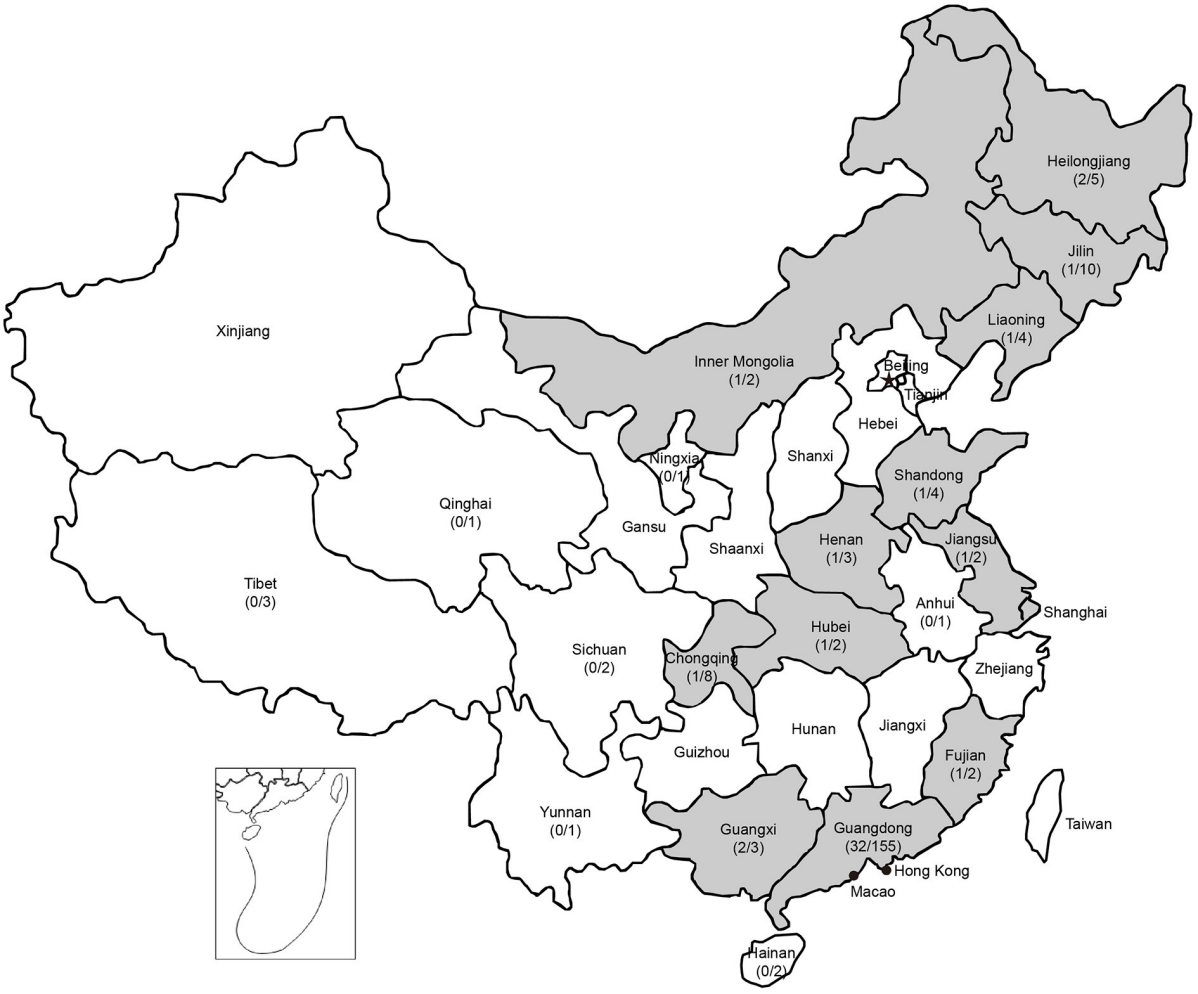
Geographic distribution of PPV8 positive samples in China. Positive rates of PPV8 infection within sampled regions are listed in parentheses. Gray background indicated the PPV8 positive regions.

To investigate the prevalence of PPV8 in China, 37 samples of different tissues (lung, kidney, spleen, liver, intestinal and lymph node) were detected as positive for PPV8 by nPCR, which were distributed over 12 regions in China with a 24-year time spanning (1998–2021) (Figure 3). There were seven positive samples from the above 55 lung tissue samples of PRRSV positive. Interestingly, three positive samples collected in 1998 were distributed in Guangdong, Guangxi and Henan provinces respectively. Sequencing and analysis of the PCR amplicons showed that 37 PPV8 strains share 97.9–100% nucleotide homology to PPV8-GDJM2021.

### Virus isolation

In this study, we attempted to isolate PPV8 using PK-15 and ST. Unfortunately, we failed to isolate PPV8 using cells and were unable to conduct follow-up studies on the pathogenicity of PPV8 in pigs.

## Discussion and conclusions

With the development of viral metagenomics research, viral metagenomic technology can not only detect sequence variation of known strains, but also detect the presence of novel viruses. RNA viruses are considered to be a major part of emerging and re-emerging viral pathogens (21). Thus RNA viruses gain more attention and research, especially for novel RNA viruses. For example, a series of novel viruses, including ephemeroviruses and birnaviruses, were identified in pigs infected by classical swine fever virus (22, 23). Few discoveries about new DNA viruses carried by pigs in recent years, to fill this knowledge gap, we tested DNA viruses from 55 lung samples across different regions in Guangdong Province by metagenomics technology.

Here, we have reported a novel parvovirus, identified in lung samples from sick pig in the Guangdong, China, by MiSeq high-throughput sequencing. According to the ICTV proposal, the difference in amino acid homology of the NS1 protein is used as the judgment standard, GDJM2021 strain was a novel virus species in the *Protoparvovirus* genus and named as PPV8-GDJM2021. The discovery of PPV8-GDJM2021 further extended the knowledge about porcine parvoviruses diversity. Although PPV8-GDJM2021 has very low genetic similarity to existing PPVs and parvoviruses of other animal, it still has relatively conserved amino acid sites of parvoviruses. Further genetic characterization of parvoviruses revealed the phylogenetic distinctiveness of PPV8-GDJM2021. The detection of clinical samples found that PPV8 is similar to other porcine parvoviruses and can be detected in multiple tissues and organs of pigs. PPV1 causes a series of conditions in pigs, and belong to the same viral genus as PPV8, but the pathogenicity of PPV8-GDJM2021 strains is currently unknown because of failure to isolate the virus in the present study.

In conclusion, this study firstly reported the genomic characterization of a novel PPV8 and improved our understanding about the genetic diversity of PPVs. Retrospective study suggests that PPV8 has wide geographical distribution and long-term existence in China, but the clinical significance and pathogenicity of PPV8 need to be resolved in the future.

## Data availability statement

The datasets presented in this study can be found in online repositories. The names of the repository/repositories and accession number(s) can be found below: OP021638.

## Author contributions

LH and WG designed, monitored the project, and wrote and revised the paper. LH, WG, YG, and GY performed all analyses. LH, CT, YG, and GY interpreted the data. YG, GY, SC, HH, JL, HZ, SL, ML, QW, and QL performed the experiments. LH, WG, and CT collected the samples. All authors contributed to the article and approved the submitted version.

## Funding

This work was supported by grants from the National Key Research and Development Program of China (2021YFD1801101).

## Conflict of interest

Authors GY, SC, and ML are employed by Guangdong Findergene Biotechnology Co., Ltd, Foshan, Guangdong Province, China. The remaining authors declare that the research was conducted in the absence of any commercial or financial relationships that could be construed as a potential conflict of interest.

## Publisher's note

All claims expressed in this article are solely those of the authors and do not necessarily represent those of their affiliated organizations, or those of the publisher, the editors and the reviewers. Any product that may be evaluated in this article, or claim that may be made by its manufacturer, is not guaranteed or endorsed by the publisher.
